# Multiphoton Phosphorescence of Simple Ketones by Visible-light Excitation and Its Consideration for Active Sensing in Space

**DOI:** 10.1007/s10895-022-02912-7

**Published:** 2022-03-17

**Authors:** Thomas de Prinse, Elizaveta Klantsataya, Georgios Tsiminis, Thomas Payten, Jillian Moffatt, Tak W. Kee, Nigel A. Spooner

**Affiliations:** 1 Institute for Photonics and Advanced Sensing (IPAS), The University of Adelaide, Adelaide, Australia; 2 Department of Chemistry, The University of Adelaide, Adelaide, Australia; 3 Defence Science and Technology Group (DSTG), Edinburgh, Australia

**Keywords:** Ketone sensing, Multiphoton, Upconversion, Phosphorescence, Space resources

## Abstract

Acetone and butanone were seen to emit blue light around 450 nm when excited in the green by a high intensity pulsed laser. The pathway of this anti-Stokes emission is believed to be multiphoton absorption followed by phosphorescence, with emission being observed in the samples at cryogenic temperatures below their melting point and not seen from either ketone in their cold liquid state. Given the widespread nature of these simple ketones in off-world bodies and their potential importance as an organic resource for Space Resource Utilization, signals which enable the identification and tracing of these materials are of use in applications from remote sensing and mapping to monitoring during extraction processes. While the excitation process has a low efficiency, the ability to use visible light for sensing of these targets has advantages over UV sources, such as the wider availability of high-powered lasers which could be utilized.

**Supplementary Information:**

The online version contains supplementary material available at 10.1007/s10895-022-02912-7.

## Introduction

Understanding the chemical make-up of off-earth bodies is crucial for expanding our comprehension of the solar system; informing both on the origins of life on earth, as well as the potential to access off-world resources to support further exploration and utilization through the system—Space Resource Utilization (SRU). A key component of this search is determining the organic compounds found in asteroids, particularly carbonaceous chondrites that contain increased quantities of organic compounds. Amongst those compounds, ketones are of particular interest as they are believed to be an essential building block in the abiotic formation [1–3] of more complex molecules such as amino acids that are detected in carbonaceous chondrite samples [4, 5].

Carbonaceous chondrites have been found to typically be comprised of 2% organic carbon compounds by weight, sometimes measured as high as 6% [6, 7]. In this family of meteorites, the simplest ketones—acetone and butanone (methyl ethyl ketone)—have been consistently detected in samples recovered on Earth [8]. The most well studied of fallen carbonaceous chondrites is the Murchison meteorite, a CM2 type chondrite containing 2.7% by weight carbon [7]. This chondrite sample has been measured to contain several hundred ppm of ketone and aldehyde compounds [9, 10], in which acetone and butanone are the most abundant ketones [1, 8–10].

Detection and identification (characterization) are both critical steps in assessing space objects as targets for resource extraction. Simple ketones themselves are a resource target, given their volatility, high oxygen to carbon ratio and chemical functionality. Effective stand-off detection would enable mapping and quantification of simple ketones where they exist in off-world bodies, most notably in C-type asteroids – asteroids of equivalent composition to recovered carbonaceous chondrites – but also potentially on planets, moons and small solar system bodies.

The standard technique for remote analysis of asteroids is reflectance spectroscopy, which detects absorption bands of the surface materials. While an increasingly popular target of this technique is the 2.7 and 3 μm bands to probe for the presence of water [11–13], most asteroids are only observed in their reflectance across 0.7 to 2.45 μm [14, 15].

While the fundamental carbonyl stretching frequency around 5.5 μm could be observed through similar measurements [16], often this spectral region is saturated with thermal emission [13, 17]. Additionally, these measurements are unable to differentiate differing carbonyl compounds. While a total carbonyl yield could be obtained, reflectance spectroscopy will always lack the ability to specifically distinguish ketones as other carbonyl compounds (such as formic and acetic acid) are also present in carbonaceous chondrites [10].

Another way in which chemicals and resources have been proposed to be sensed in space is through fluorescent imaging [18–20]. Fluorescence or phosphorescence, where it exists, can be bright and highly indicative of a particular target [21, 22], enabling both qualitative and quantitative analysis to be done remotely [23–25].

In this study, a high intensity pulsed visible light laser is used to induce shorter wavelength (multiphoton upconversion) emissions from simple ketones in their solid state at cryogenic temperatures. The ability to undertake multiphoton upconversion sensing using high-powered visible wavelength lasers has the potential to create another mode of active sensing for small observational crafts that are mapping or searching for resources. Utilizing visible lasers for excitation has advantages over contemporary ultraviolet sources, such as a wider availability of available high power laser sources, which could see them being used despite the low excitation efficiencies associated with this multiphoton absorption step.

## Experimental Design

**Fig. 1:**
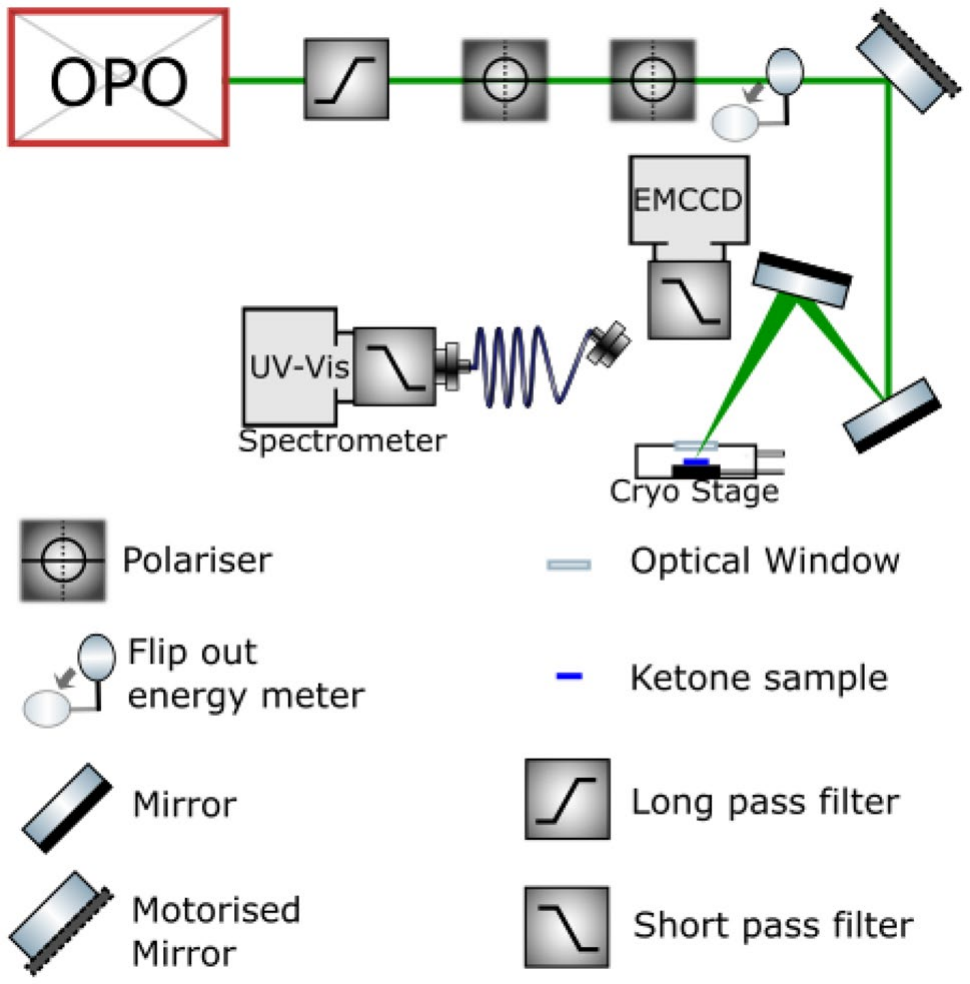
The optical pathway for the excitation and emission detection for the samples in the cryogenic stage

The optical setup is depicted in Fig. 1. For the excitation scan and emission spectra, the sample was excited by an optical parametric oscillator (OPO), driven by a flashlamp-pumped 5 ns 1064 nm Nd:YAG laser at 10 Hz (OPOTEK ‘Radiant’ HE 355). The temperature and power dependence were measured in a similar setup utilizing a different OPO, driven by a flashlamp-pumped 5 ns 1064 nm Nd:YAG laser at 20 Hz (OPOTEK ‘Opolette’ HE 355). Lifetime measurements were conducted using the Opolette laser and a Hamamatsu R928 photomultiplier tube through a monochromator.

The direct output from the Radiant OPO laser contained several parasitic beams that were removed before reaching the sample. Two short pass filters (Semrock BrightLine, 715 nm blocking edge) were used to remove both pump light at 1064 nm and parasitic idler wavelengths in the range of 800−1250 nm. These filters also served to remove leaked 355 nm pump light from the beam. Two Glan-Laser calcite polarizers were used to further remove parasitic idler laser emissions including 532 nm pump light from the beam, such that no other laser lines other than the selected 500 nm to 670 nm signal wavelength range was incident on the sample. The high spectral purity of the laser source allowed confidence that the observed sample emissions were not being induced by parasitic laser lines, such as leaked UV light.

The energy of the laser was quantified during each measurement with a flip-out energy meter, averaged over 10 pulses. The measured emission intensity was then normalized to the changing intensity of the laser as it was scanned over the excitation range (Fig. S1).

The beam spot was maintained on the sample by a motorized mirror, which made small pre-determined adjustments to compensate for any drift in the beam position as the OPO scanned across different signal wavelengths.

A fiber-coupled Princeton Instruments Acton SpectraPro Sp-2300 Spectrometer with a cooled PIXIS 100 CCD was used to collect emission spectra. To improve the signal to noise ratio, the CCD pixels were hardware binned in groups of 4. The spectrometer was wavelength calibrated with a mercury gas discharge lamp.

Two short-pass filters (Semrock BrightLine, 532 nm blocking edge) were placed in the fiber collection optics to prevent diffraction grating artefacts from the longer wavelength excitation laser.

The sample was imaged using a Princeton Instruments ProEm 1024BX3 Electron multiplied CCD (EMCCD) with a 60 mm macro lens and two short pass filters (Semrock BrightLine, 498 nm blocking edge). Sample imaging with the EMCCD confirmed that the detected light was originating from the sample and also ensured that the laser beam spot did not drift away from the sample during the scans.

Temperature was maintained at 163 ± 0.2 K using an Instec HCS622V thermal stage, cooled by liquid nitrogen. A nitrogen atmosphere was maintained above the sample by the stage system during the excitation process, with the sample undergoing several freeze–thaw cycles under the nitrogen atmosphere prior to analysis. The nitrogen gas was also utilized to prevent moisture from condensing on the stage’s optical window during experiments.

Acetone and butanone (2-butanone, methyl ethyl ketone) were spectroscopic grade (Sigma Aldrich) and used neat.

## Results

**Fig. 2:**
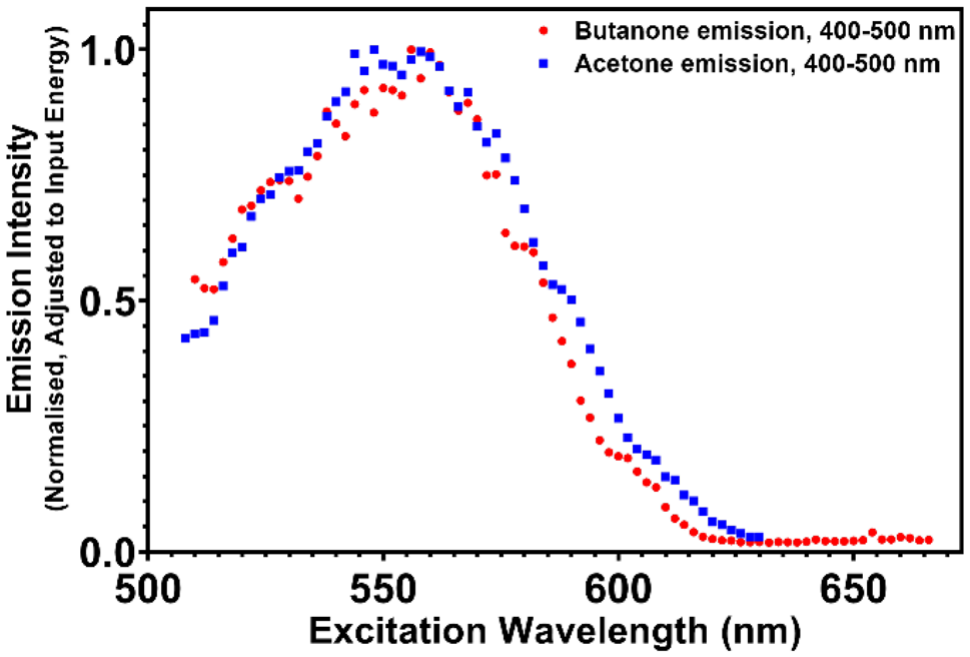
Excitation spectra of acetone and butanone, frozen at 163 K. Emission was collected from 400–500 nm

**Fig. 3:**
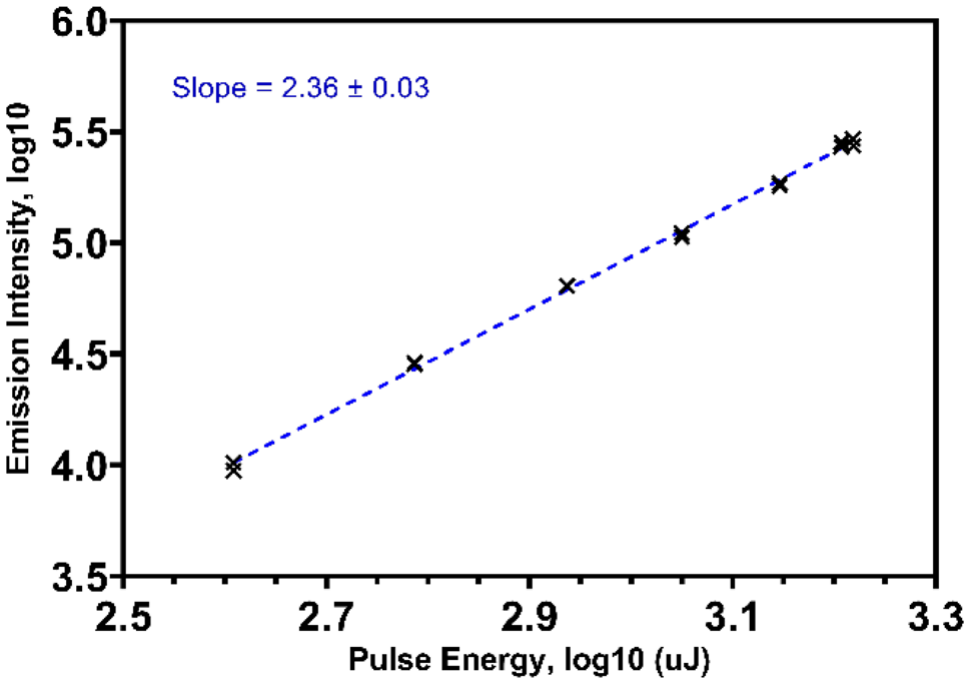
Power dependence plot of acetone at 163 K, excited by 545 nm pulses. Pulse energy was varied by rotation of a Glan-Laser polariser

Acetone and butanone have no notable absorbance bands in the visible region, yet the excitation spectrum of both frozen acetone and butanone exhibits a peak at 555 to 560 nm for emission in the blue region (Fig. 2). This excitation peak corresponds to half the energy of the peak **S**_**0**_ → **S**_**1**_ absorbance in the UV region at 275 nm for both materials [26–28]. The excitation pathway is therefore multiphoton absorption, in which the molecule is promoted to the same excited state as with UV excitation through the ‘simultaneous’ absorption of two photons of green light via a virtual state. This non-linear effect with low transition probability can be observed due to the high laser pulse intensities used [29]. The non-linear dependency is demonstrated in Fig. 3 with a slope of over 2. A power dependency slope of this magnitude shows the process requires twice as many photons for excitation than for emission, as is the case for the green to UV multiphoton absorption.

**Fig. 4:**
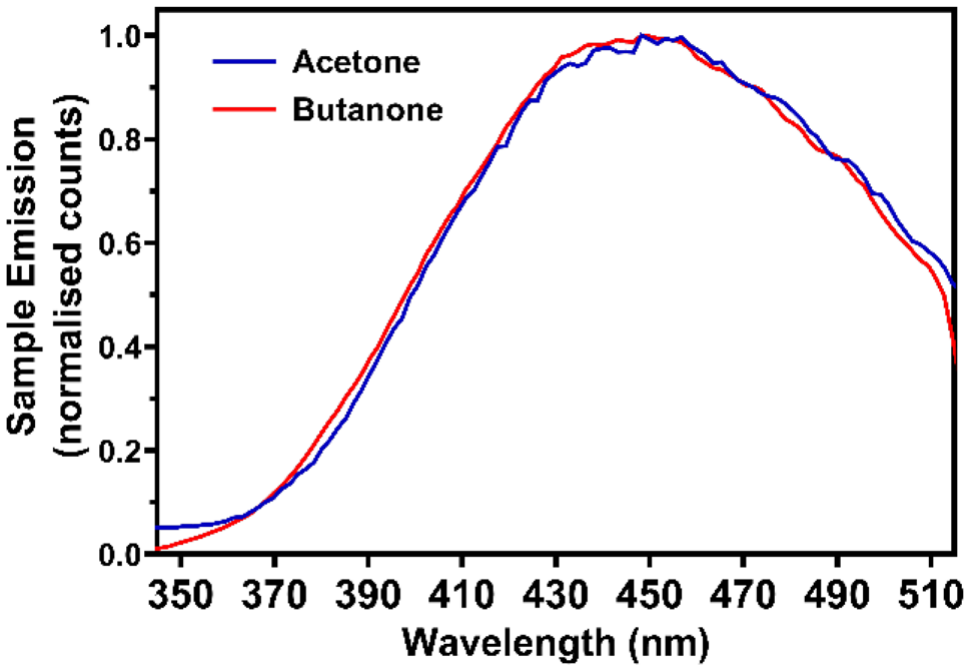
Emission from acetone and butanone at 163 K when excited at their peak excitation wavelength (556 nm). Spectra collected through two 532 SP filters, and normalised to the peak value

The emission spectra for both samples are broad and peak at approximately 440 nm, which are presented in Fig. 4. This emission profile corresponds well to previously recorded phosphorescence spectra of liquid acetone recorded at room temperature under UV excitation [30–32], where the peak emission occurs at 455 nm. Only a minor blue shift is seen on comparison between room temperature and frozen samples, which reflects the lack of shift seen in the main excitation peak in Fig. 2, situated at twice the wavelength of the room temperature UV absorbance peak.

The lifetime of the multiphoton acetone emission was measured to be 830 ± 10 µs at 123 K (Fig. S2). This corresponds well to previously measured acetone phosphorescent lifetimes of 700 µs seconds at 175 K and 1.0 ms at 77 K, excited in the UV [30]. An excited state lifetime of this magnitude confirms that the emission signal being observed is phosphorescence.

**Fig. 5:**
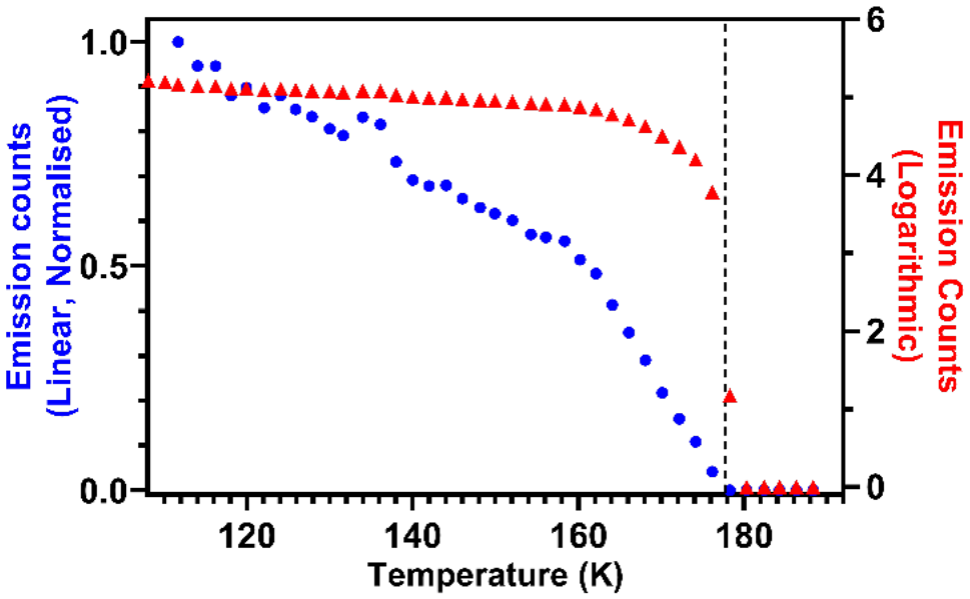
Emission intensity from acetone excited at 545 nm as temperature is increased, shown in both linear (blue, left axis) and logarithmic (red, right axis) representation. The melting point of acetone is highlighted by the dashed line at 177.7 K

Multiphoton phosphorescence was not observed in either sample above their melting point. Figure 5 shows this loss of signal in acetone as the melting point is reached, best seen in the logarithmic scale. The emission intensity decreases as a function of temperature, but interestingly a change in the rate of decrease was seen at approximately 160 K, best seen in the linear scale of Fig. 5. This change was present regardless of whether the temperature was increased or decreased over this range during the experiment. Changes in the dielectric constant and heat capacity of solid acetone over this range [33] have been attributed to shortening of intermolecular carbonyl distances and increased electrostatic interactions [34], which may also play a role in the yield of the emission pathway. Additionally, a metastable orthorhombic C-centered phase has been observed just below the melting point of acetone through crystallography [34] and neutron scattering measurements [35]. Therefore, the measured change in the temperature dependence of emission intensity at 160 K may also be due to a phase change in the material back to the stable primitive orthorhombic phase.

## Discussion

The similarities of the emission and excitation profile of acetone and butanone do not permit discrimination between the two, but demonstrate the capability to act as an indicator of the presence of these simple ketone molecules. In both molecules, the S_0_ −> S_1_ transition is a forbidden n −> π* transition, where poor orbital overlap and orbital symmetry therefore result in a low molar absorptivity of approximately 10–15 L/mol cm [36, 37]. The value slightly increases to approximately 20 L/mol cm upon cooling to 77 K [37].

The fluorescence quantum yield of acetone at room temperature in fluid solution is reported to be approximately 0.01 [30, 38]. Phosphorescence yields in room temperature degassed solvents were less than 0.003 [37] which increase at 77 K (with acetone as a rigid organic glass) to approximately 0.03 [30]. The small energy gap between the S_1_ excited state and the T_1_ manifold [37] as well as the forbidden nature of the S_1_ −> S_0_ transition makes intersystem crossing (S_1_ −> T_1_) extremely favorable, approaching a φ_ST_ value of 1 [39] independent of temperature [30]. Because of this, phosphorescence is known to dominate fluorescence in the condensed state [31]. Lifetimes of excited states and the fluorescence to phosphorescence ratio is significantly different for acetone in the vapor state [27, 40].

The phosphorescence detected from the frozen ketone sample was weak in comparison to the high intensity input laser pulses, even considering the low reported quantum yields. The multiphoton process is inefficient due to the low absorptivity at the input light wavelength, therefore resonance enhancement is expected to be minimal [29, 41]. This can be noted as well in Fig. 3, which shows no evidence of the excitation process becoming saturated despite the use of high intensity pulses.

A multiphoton absorption step therefore places a constraint on the efficiency of the detection of ketones by fluorescence or phosphorescence. A multiphoton process has an identical emission pathway and quantum efficiency in comparison to the single photon process once the S_1_ excited state is reached [42], so the true quantum yield of the multiphoton process remains the reported value of *ϕ*_phos_ = 0.03. Poor absorption of the visible light is expected to be accountable for several orders of magnitude loss in efficiency in regard to emission signal produced for the amount of laser intensity used during excitation [42].

## Application to Off-world Bodies

Detection of the ketone samples was possible due to low temperatures enhancing and enabling the emission processes. In this way, emission signatures that are absent under room temperature conditions can be observed from the cold organic solids. This is of particular relevance to off-world bodies, which usually have substantial regions with a surface temperature below the freezing point of the organic sensing targets [13, 43], even at distances as short as 2 AU [44].

**Fig. 6:**
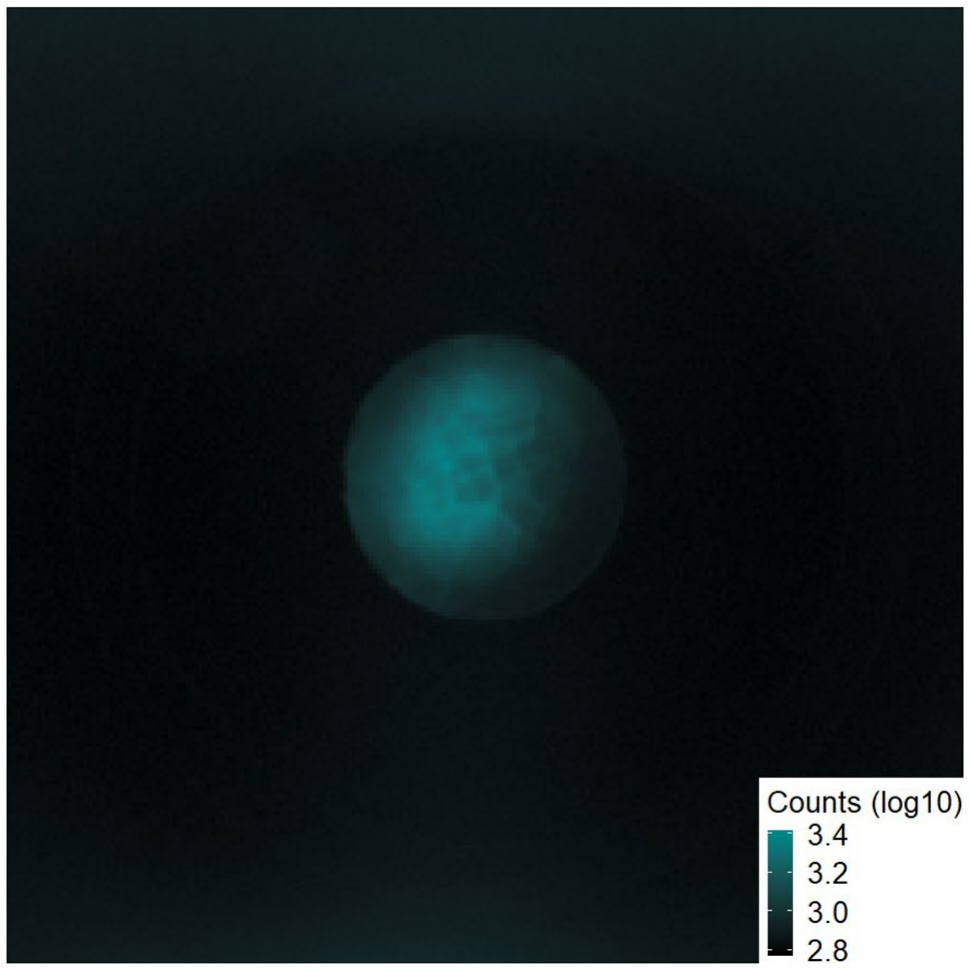
EMCCD image of butanone through the temperature stage window. Excitation at 532 nm, imaging through two 498 nm short pass filters. The optical window is 1 inch in diameter

Fluorescence and phosphorescence in organic molecules including ketones are readily induced with wavelengths in the UV, however laser wavelengths in the visible range are much more accessible for use in space. Excitation sources producing light in the green, such as diode pumped fiber lasers, are capable of high intensities while remaining lightweight and relatively simplistic [45–47]. An excitation system which has been suggested for use off-world utilizes the frequency multipliers of a 1064 nm laser [48]. While the 266 nm fourth harmonic can be made available for sensing through this system, our research shows that the 532 nm second harmonic also can play the key role, with signal emissions being blue shifted from the laser line (Fig. 6). Low efficiency processes on the sensing target such as a multiphoton absorption step can be overcome through the much-increased intensity readily available at visible laser wavelengths such as the 532 nm harmonic.

Further work is required to determine how well this technique could enable selective sensing of ketones against other carbonyl molecules, such as formaldehyde [49], carboxylic acids [50], and carboxylate salts [51].

The two similar molecules of acetone and butanone could not be differentiated in this work by luminescence sensing, with many similar molecules likely to be indistinguishable in regards to their excitation and emission properties. Changes to functional groups and larger variations in molecular structure however are expected to produce discernable alterations to the spectral and temporal characteristics of the emission. Different peak excitation wavelengths could also allow for some degree of selective excitation of particular organic molecules. Remote differentiation between similar carbonyl molecules or selective detection of a carbonyl target would prove to be a tremendous advantage that is currently unmatched by other remote sensing techniques.

## Conclusion

Light emission was induced from frozen acetone and butanone samples with a pulsed laser in the visible region, believed to be multiphoton absorption followed by phosphorescence. This emission signature was not observed in cold liquid samples but was readily observed once the samples had frozen, the state they would occupy in many off-world small solar system bodies. While the overall efficiency of this excitation process is low, using visible lasers to drive multiphoton fluorescence or phosphorescence should be viable due to the availability of high powered, robust visible light sources. The ability to selectively target ketone species without the use of UV is a great advantage and adds to the rapidly progressing field of material-specific sensors helping to explore and understand the solar system.

## Supplementary Information

Below is the link to the electronic supplementary material.

Supplementary file1 (DOCX 444 KB). OPO laser pulse energy relating to figure 2, as well as acetone emission lifetime data and exponential fit.

## Data Availability

The unprocessed datasets generated during this study are not publicly available due to funding agreements with the Commonwealth Government Department of Defence, but may be made available from the corresponding author on reasonable request.

## Code Availability

Code used to process the raw data is not publicly available.
