# Supplementary material for: Multiphoton Phosphorescence of Simple Ketones by Visible-light Excitation and Its Consideration for Active Sensing in Space
Source: J Fluoresc. 2022 Mar 17;32(3):1051–7. doi: 10.1007/s10895-022-02912-7 (PMC9095556; doi:10.1007/s10895-022-02912-7)
Supplement: Supplementary file 1 — Supplementary file1 (DOCX 444 KB). OPO laser pulse energy relating to figure 2, as well as acetone emission lifetime data and exponential fit. [file 10895_2022_2912_MOESM1_ESM.docx]

**Multiphoton phosphorescence of simple ketones by visible-light excitation and its consideration for active sensing in space**

**Supplementary Information**

**Fig. S1** Pulse energy of the OPO laser during the excitation scans of the two ketone samples, depicted in Figure 2.

**
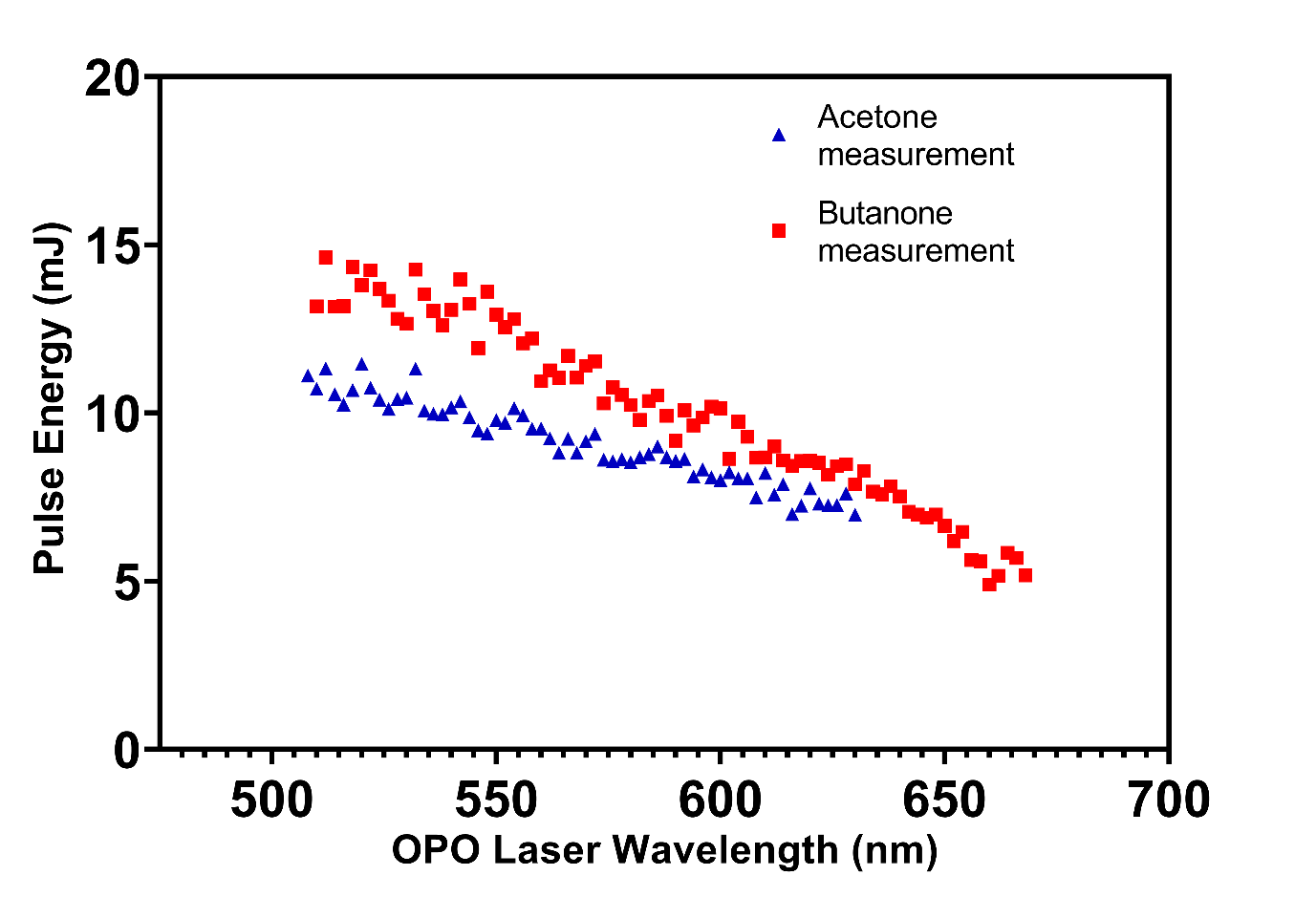
**


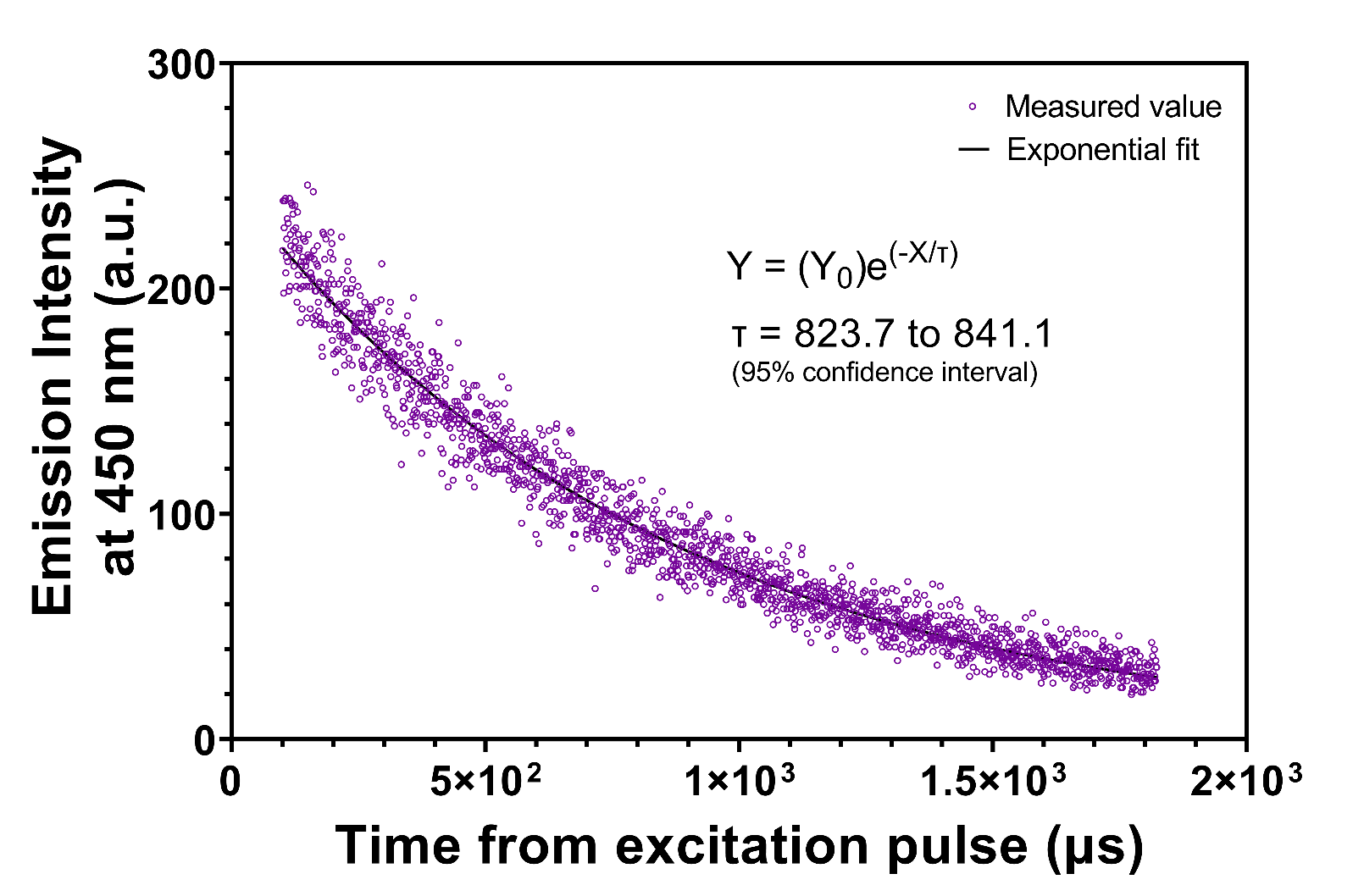


**Fig. S2** Lifetime measurements of solid acetone at 123 K, excited by 560 nm laser light with detection at 450 nm. Fitting to a single exponential (equation given on the chart) gives an lifetime value (τ) of 830 ± 10 μs.
